# Towards food systems transformation—five paradigm shifts for healthy, inclusive and sustainable food systems

**DOI:** 10.1007/s12571-021-01221-4

**Published:** 2021-10-15

**Authors:** Ruerd Ruben, Romina Cavatassi, Leslie Lipper, Eric Smaling, Paul Winters

**Affiliations:** 1 Professor Impact Analysis of Food Systems, Wageningen University and Research, 2502LS The Hague, PO Box 29703, Wageningen, the Netherlands; 2 Senior Economist. International Fund for Agricultural Development, Via Paolo di Dono, 44, 00142 Roma, Italy; 3 International Fund for Agricultural Development (IFAD), Senior Consultant Rural Development Report, Wageningen, the Netherlands; 4 Senior Researcher. Wageningen Environmental Research, PO Box 47, 6700 AA Wageningen, the Netherlands; 5 Keough-Hesburgh Professor of Global Affairs in Keough School of Global Affairs, University of Notre Dame, 4027 Jenkins Nanovic Halls, Notre Dame, IN 46556 USA

**Keywords:** Food systems transformation, Paradigm shift, Trade-offs, Synergies, Governance

## Abstract

Food systems must serve different societal, public health and individual nutrition, and environmental objectives and therefore face numerous challenges. Considering the integrated performances of food systems, this paper highlights five fundamental paradigm shifts that are required to overcome trade-offs and build synergies between health and nutrition, inclusive livelihoods, environmental sustainability and food system resilience. We focus on the challenges to raise policy ambitions, to harmonize production and consumption goals, to improve connectivity between them, to strengthen food system performance and to anchor the governance of food systems in inclusive policies and participatory institutions. Taken together, these shifts in paradigms shape a new discourse for food system transformation that will be capable to respond to current and future policy challenges.

This Series on Food System Transformation published in *Food Security* looks at challenges, prospects, and strategic options for transforming food systems to become:

- *Healthy and nutritious –* providing nutritious and affordable diets for good health.
- *Inclusive –* enabling a decent living for all stakeholders in the food system so no-one is left behind.
- *Environmentally sustainable –* consuming and producing food respecting planetary boundaries.
  Meeting these three objectives in transformed food systems also implies that food systems have to be:
- *Resilient –* ensuring that people can access food and protect their livelihoods when food systems are hit by extreme events or market shocks and political instability or conflicts.

Around the world, imbalances and disconnected food markets and governance are generating undesirable trade-offs between (i) food supply, (ii) consumption patterns, (iii) nutrition, (iv) livelihoods, and (v) the environment. These are key concerns for the 2021 UN Food Systems Summit (UNFSS) and are discussed in the 2021 Rural Development Report (IFAD, 2021) *Food System Transformations for Rural Prosperity* by the International Fund for Agricultural Development (IFAD). Current trends in poverty, malnutrition and climate change reflect widespread failures in food systems. To address the trade-offs and make progress in all areas, we need a clear view of how food systems are organized and how different stakeholders interact.

Our special concern are poor people, in rural but also in urban areas. What will food system changes mean for employment and small-scale producers? What kind of food system transformations can improve nutrition? What factors drive transformation of food systems in less developed countries? Can these drivers interact in ways that will promote healthy, inclusive and sustainable food systems? Will the resulting food system respect planetary boundaries, and improve the state of world ecosystems? What policy instruments can support such transformation processes? What is the role of governance and what kind of governance is needed to ensure such transformations?

Two constituencies are at risk of being left behind in the transforming food systems. On the one hand, about half a billion small-scale self-employed rural producers including farmers, herders and fishers accounting for three billion people globally (woodhill et al., 2020), and some two billion men and women engaged in the informal economy that are currently too poor to have a secured economic access to basic food requirements (Global Nutrition Report, 2020; International Labour Office, 2018). On the other hand, healthy diets are now out of reach for at least three billion people in the Global North and the Global South alike (Herforth et al., 2019; Hirvonen et al., 2020). The Covid-19 crisis has substantially increased this number (Swinnen an McDermott, 2020). In many cases, high relative food prices together with lower incomes largely explain the prevalence of undernutrition and overweight. How can poverty and malnutrition be addressed through food system changes that harness opportunities while avoiding trade-offs?

Food systems include all elements and activities related to food production, processing, distribution, preparation, consumption and disposal – including market and institutional networks for their governance – and they include the outcomes of these elements for health, livelihoods and the environment. The analytic framework of HLPE (2017) underlies this definition of food systems (Fig. 1) and distinguishes linkages and feedbacks among three key food system areas:

- *Drivers* –factors external to food systems per se, including population growth and urbanization, technological development, climate change, trade and economic growth.
- *Components* – elements directly related to food production and value chains (processing and distribution), diets (preparation and consumption) or food environment (markets and institutions).
- *Outcomes –* healthy diets, livelihood, well-being including equity and inclusiveness, and system sustainability as well as resilience, including to climate change.

**Fig. 1:**
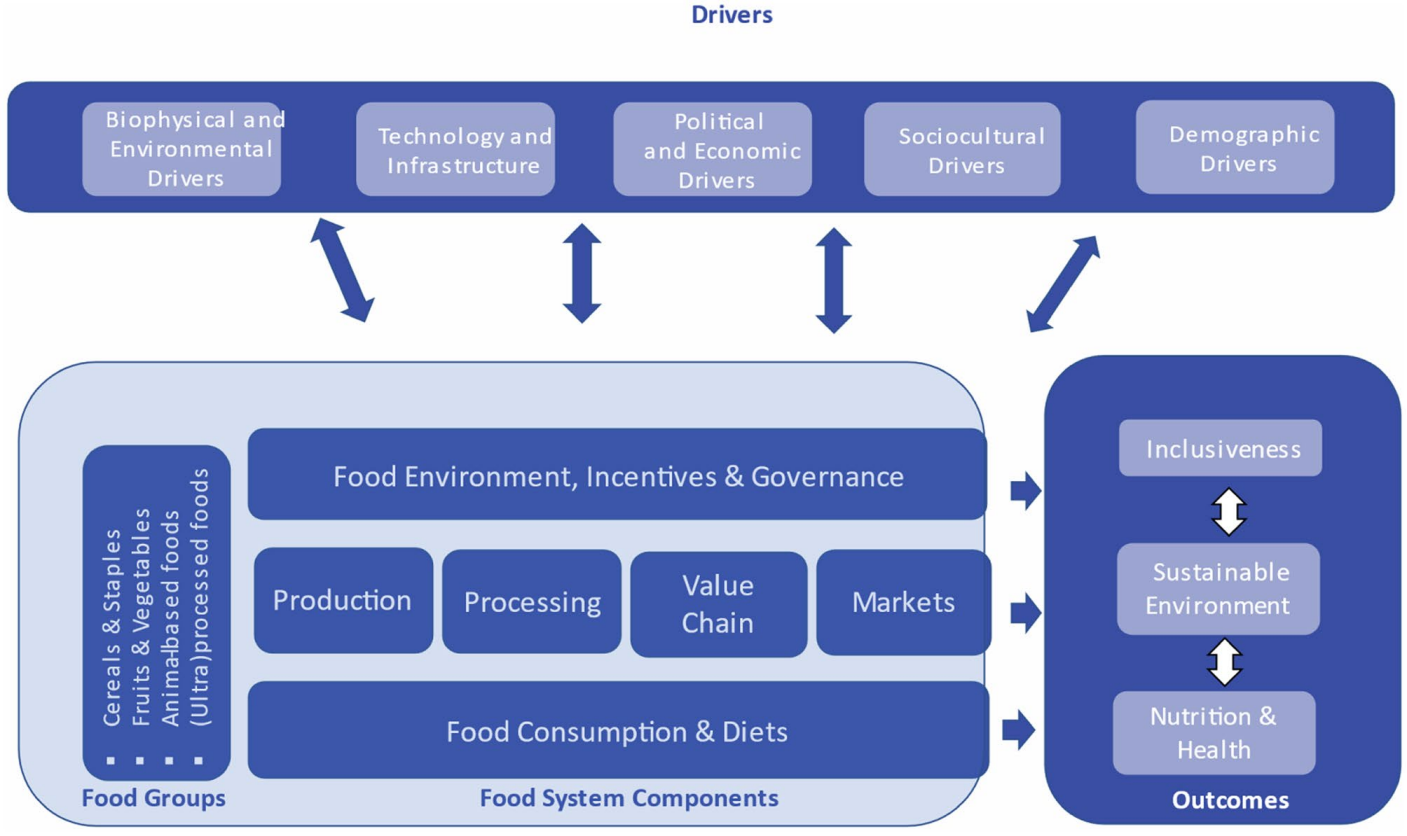
Food systems analysis framework ( adapted from HPLE, 2017)

The food environment plays a central role in the food system framework because incorporates all the infrastructure, public and private, institutional regimes and governance frameworks that guide food availability, accessibility, quality, safety, sustainability, reliability and affordability (Caspi et al., 2012; Herforth & Ahmed, 2015; Turner et al., 2018). There are structural imbalances and disconnects that prevent the delivery of desired outcomes for nutrition, inclusion and environmental sustainability.

Opportunities for food system transformation depend largely on the scope for improving potential agricultural productivity by reducing the gaps between actual and achievable yields (van Ittersum et al., 2016), and by changes in land use from calory-rich to nutrient-dense activities (horticulture, animal-based activities), as well as opportunities for improving incomes (Arslan et al., 2021). The latter is supported by shifting labour from agriculture to non-farm employment in trade, manufacturing and service sectors. Both processes have significant effects on farm size, on natural resource use, and on rural and urban incomes. The transition towards more inclusive and resilient food systems requires radical changes in all food system components: production, consumption, trade and governance.

The articles included in the Series highlight five paradigm shifts that are needed to better understand the requirements, scope and implication of strategies towards food system transformation:

1. **Raise Ambitions: from food security to food system resilience**
  Much attention has been given to strategies for improving food security at individual, regional and national level. This goal may be reached through a combination of raising productivity, improving returns on labour and strengthening market connections (Tendall et al., 2015). However, it is increasingly recognized that long-term food security cannot be reached without improving the resilience of food systems (Lipper et al., 2021). This requires that producers and consumers are able to adapt to unexpected changes in the (natural and policy) environment, through diversification strategies for livelihoods, diets and markets that enable flexible and timely responses to global change. To ensure resilience and a functional link with the circular economy, these strategies must also contribute to the long-term satisfactory functioning of food systems in providing nutrition, environment and livelihood benefits in the process of producing, supplying, consuming and disposing/recycling foods at varying levels and across different food system types.
  The main rationale for the growing interest in food system transformation is related to the growing recognition that the multiple problems of poverty, malnutrition, environmental degradation and climate change are combined, and cannot be ‘fixed’ with single interventions, but instead need a fundamental change in the dynamics of food systems (Giller et al., 2021a, b). In response to the triple challenge of malnutrition – hunger, micronutrient deficiencies and overweight – comprehensive strategies for supporting availability, access, safety, affordability and desirability of food need to be defined.
  Agricultural production from the large majority of smallholder producers creates insufficient marketable surplus to nourish the growing urban population (Giller et al., 2021a; Barthel et al., 2019). Moreover, the growing demand for food also supports a further transformation in the agrarian structure, with an increasing number of midsize farms and the reduction of farm size operated by smallholders (Giller et al., 2021b; Jayne et al., 2016; Tschirley et al., 2015). Addressing the tension between improving livelihoods of smallholders and ensuring adequate and nutritious food supplies will be an important aspect of enhancing resilience of the overall food system in the next decades.
2. **Harmonize Goals: combine efficient production with affordable nutrition, inclusive livelihoods and environmental sustainability**
  Overcoming the current trade-offs in food system performance requires decisive efforts to reach a new balance between food production and consumption in agro-ecosystems that are becoming more fragile (Terwisscha van Scheltinga et al., 2021). Harmonizing healthy diets with sustainable food production is certainly feasible but requires clear policy guidance (Willett et al., 2019). A main reason to advocate the transformation of food systems—as opposed to single-target interventions—lies in the need to simultaneously improve nutrition, inclusiveness and environmental sustainability (van Berkum & Ruben, 2021). Better connections between agricultural production and food value chains with diets, human health, livelihoods and agro-ecosystems requires stakeholder coordination and policy bargaining on harmonization of goals (Fanzo et al., 2021; Webb et al., 2020).
  While efficient, reliable, and sustainable food production remains critical, a sole focus on agricultural productivity has led to some unintended and unwelcome outcomes in addition to being insufficient. While productivity growth has contributed to lifting many people out of poverty, progress has been uneven across and within countries (Pingali., 2015). Moreover, the way intensification has been carried out has raised environmental concerns (Giller, 2021a) whereas 37% contribution to GHG emissions coming from food system calls for a dramatic reduction to comply with Paris agreement and mitigation needs (Lipper et al., 2021). A focus that would solely consider increasing yields of staple crops may result in more affordable calories for consumers, but not necessarily adequate and affordable nutrition based on nutrient-dense and diverse diets required to address malnutrition (Brouwer et al., 2021).
  Reinforcing food system resilience implies attention to diversification. Diverse diets will improve nutrition and health – only if such diverse food supply comes with increased affordability and accessibility for nutrient-dense foods (Brouwer et al., 2021; Pingali, 2015). Diversifying food production can improve rural livelihoods while supporting biodiversity and landscape management of natural resources (Bommarco et al., 2013).
  Lastly rural livelihood can be improved when inclusiveness is ensured. Gender and inequality in food systems are strongly related. Women are actively involved in food systems, yet their contributions to food systems are often not recognized. By and large, women face constraints that prevent them from engaging in food systems on terms that are equitable and fair. Women’s empowerment is essential to achieving the objectives of (1) healthy, safe, and diverse diets that meet the nutrient requirements of all household members; and (2) inclusive food systems that engage smallholder farmers in food production and ensure affordable access to diets by disadvantaged groups of consumers. This also applies to indigenous people of which nearly three quarters live in rural areas and whose wellbeing is critical for the sustainable management of a large share of the world’s natural resources.
3. **Improve connectivity: from modular exchange to midstream interlinkages**
  Food system resilience can be reinforced by linking rural and urban constituencies (De Bruijn et al., 2021) and by increasing non-farm and off-farm employment opportunities that absorb surplus labour. Investments in midstream small and medium enterprises for local processing, storage and retail provide important new sources of employment, support value added creation and create opportunities for circular resource use (Reardon et al., 2021; Felicity et al., 2016). Linking farmers and consumers to reliable and transparent informal and formal markets offers the potential of contributing to better access to affordable and healthy diets and reinforces nutrition, inclusion and sustainability – as well as greater stability of food supply (van Berkum, 2021). Improved diets in turn generate substantial welfare and health benefits that may become an additional source of pro-poor growth.
  Different types of connections influence food system performance. Tailoring food supply (production) to food demand (consumption) is heavily influenced by interactions between technology and behaviour (Ruben et al., 2019). The available infrastructure for transport and communication offers spatial connections for local and interregional trade between rural and urban areas (De Bruijn et al., 2021; Proctor & Berdegue, 2016). Communication infrastructure and smart ICT devices can become particularly helpful for timely distribution of information, thus enabling the responsiveness of food system stakeholders to potential shocks (Ceccarelli et al., 2021).
  These spatial linkages partly coincide with vertical sectoral linkages between supply chain actors that determine to a large extent the value added distribution and the incentives for food system upgrading (Liverpool-Tasie et al., 2020; Reardon et al., 2021). The dynamics of midstream agents in charge of transport, storage, processing, and retail strongly determines the responsiveness of food systems (Reardon et al., 2021). Social and environmental externalities of food system operations should be considered in the process of price formation, taking into account principles of living income, health and climate change (Alho et al., 2021). Sustaining connectivity also asks for supply chain relationships that consider the equitable distribution of value added amongst producers, traders and retailers (Waarts et al., 2021).
4. **Strengthen responsiveness: from linear agri-food value chains to circular food systems**
  Food systems transformations are interactive processes that need adaptive capacity to be able to adequately respond to unexpected challenges. The evolution of food systems is not a linear process and multiple trends appear simultaneously (Dengerink & Guijt, 2021). Different types of food systems have diverse and specific pathways for providing healthy, affordable, safe and sustainable diets – and thus need tailor-made solutions (Garbero et al., 2021). However, across all food system types, moving to circular systems based on resource recycling serves the purpose of enhancing responsive and efficient resource use.
  Promoting circular food systems is based on a thorough understanding of major leakages. Reduction of post-harvest losses and waste (PHL) is critically important and requires investment in physical infrastructure and food management (Stathers et al., 2020). Recycling and re-use can contribute to better material balances (Martins de Oliviera et al., 2021). Shelf-life of many perishable products can be extended if upstream drying or fermentation practices are applied to reinforce food integrity downstream in the food system (Schoustra et al., 2021; Adeyeye, 2017). Strategies for improving local indigenous foods that rely upon resource recycling can also be important in supporting youth employment and female entrepreneurship (Schoustra et al., 2021).
  Since global food production is the biggest driver of environmental degradation (Clark et al., 2019; Poore & Nemecek, 2018), special attention is given to strategies towards optimal use of biomass from crop-based systems, opportunities for reducing pressure on forest and biodiversity, and the possibilities for improving feed-food conversion and circularity within livestock systems (Oosting et al., 2021).
5. **Anchoring governance: from targeted incentives to integrated and comprehensive food system governance**

Policies, investments and innovations in the agricultural sector have focused on interventions to alter incentives among producers in an effort to boost production or to enhance value chain efficiency. They rarely looked beyond immediate production and profitability concerns of producers of individual commodities. This governance approach can lead to a myriad of unintended consequences within food system since it fails to incorporate objectives such as inclusion, nutrition and sustainability, and neglects consumer and citizen interest in the food system beyond the farm and immediate value chain. The current governance system and focus on Ministries of Agriculture and related stakeholders is unlikely to resolve these issues and a broader approach and thinking is needed.

Strategies for promoting inclusive food system transformation require a sound anchoring of policy incentives, public investments and institutional and business innovations. Given the wide variety of resources and livelihoods, and the diversity in cultures and markets, food system transformation can only be orchestrated through policy experiments based on a common agenda, capacity building for all stakeholders involved and transparent feedback mechanism (Boogaard et al., 2021). Such combination of technical, institutional and behavioural innovations for linking healthy consumption to sustainable food supply needs to be supported by broad participation of all relevant stakeholders.

Strengthening food system interactions is critical to guarantee that linkages between production and consumption and between rural and urban constituencies are sufficiently inclusive. It is generally recognized that women empowerment is critical to overcome inequalities in dietary intake. In additional to pre- and post-natal health care, providing resources and education to women reinforces their voice and bargaining power and contributes to the reduction of poverty gaps and a more equal distribution of food (Quisumbing et al., 2021).

## Outlook and policy challenges

Engaging stakeholders in food system transformation processes is by no means an easy challenge. It requires a fundamental change in our thinking and a paradigm shift at all levels, ranging from food system analysis and diagnostics to food systems policies and governance. We need to look beyond some selective activities that intend to ‘repair’ local failures towards a full overhaul of the dynamics of global food systems and the interactions between food system stakeholders.

**Fig. 2:**
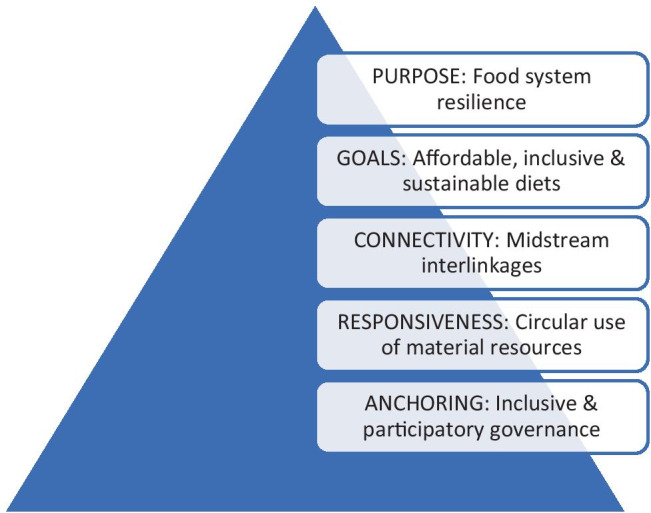
Food system transformation pyramid

Coherent policies for more inclusive and nutrient-sensitive investment are required to improve food quality, food safety and food system sustainability for broad categories of stakeholders. Both market incentives and public regulation are necessary to support connectivity and to enhance responsiveness. In addition, anchoring food system change in policies, institutions and culture requires that due attention is given to social norms and differences in power (Fig. 2).

The five necessary paradigm shifts outlined in this Series on Food System Transformation provide a wider understanding of the requirements and scope of food system transformation strategies that are discussed during the 2021 UN Food Systems Summit (UNFSS). The urgency for accelerating food system transformation—motivated by growing malnutrition in several parts of the world and rising environmental and social costs related to unhealthy and unsafe diets – makes it is imperative to address these challenges in a comprehensive manner.
